# {2-[2-(Isopropyl­amino)­ethyl­imino­meth­yl]-5-meth­oxy­phenolato}(thio­cyanato­-κ*N*)nickel(II)

**DOI:** 10.1107/S1600536810027236

**Published:** 2010-07-14

**Authors:** Si-Yu Yue, Jiu-Fu Lu

**Affiliations:** a School of Chemistry and Environmental Science, Shaanxi University of Technology, Hanzhong 723000, People’s Republic of China

## Abstract

In the title mononuclear complex, [Ni(C_13_H_19_N_2_O_2_)(NCS)], the Ni^II^ ion is coordinated by one phenolate O atom, one imine N atom, and one amine N atom of a 2-[2-(isopropyl­amino)­ethyl­imino­meth­yl]-5-meth­oxy­phenolate Schiff base ligand, and by one N atom of a thio­cyanate ligand, forming a slightly distorted square-planar geometry.

## Related literature

For background to the study of complexes with Schiff bases, see: Hamaker *et al.* (2010 ▶); Wang *et al.* (2010 ▶); Mirkhani *et al.* (2010 ▶); Liu & Yang (2009 ▶); Keypour *et al.* (2009 ▶); Adhikary *et al.* (2009 ▶); Peng *et al.* (2009 ▶). For related nickel complexes, see: Wang & Wei (2006 ▶); Wang (2007 ▶); Arıcı *et al.* (1999 ▶).
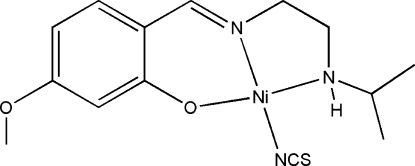


## Experimental

### 

#### Crystal data

[Ni(C_13_H_19_N_2_O_2_)(NCS)]
*M* *_r_* = 352.09
Monoclinic, 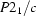

*a* = 12.5653 (10) Å
*b* = 11.5197 (9) Å
*c* = 12.6916 (10) Å
β = 119.393 (4)°
*V* = 1600.6 (2) Å^3^
*Z* = 4
Mo *K*α radiation
μ = 1.35 mm^−1^
*T* = 298 K
0.25 × 0.23 × 0.22 mm

#### Data collection

Bruker APEXII CCD area-detector diffractometer
Absorption correction: multi-scan (*SADABS*; Sheldrick, 1996 ▶) *T*_min_ = 0.729, *T*_max_ = 0.756
9225 measured reflections
3458 independent reflections
2494 reflections with *I* > 2σ(*I*)
*R*_int_ = 0.026

#### Refinement

*R*[*F* ^2^ > 2σ(*F* ^2^)] = 0.058
*wR*(*F* ^2^) = 0.160
*S* = 1.07
3458 reflections
196 parameters
1 restraint
H atoms treated by a mixture of independent and constrained refinement
Δρ_max_ = 0.75 e Å^−3^
Δρ_min_ = −0.60 e Å^−3^

### 

Data collection: *APEX2* (Bruker, 2004 ▶); cell refinement: *SAINT* (Bruker, 2004 ▶); data reduction: *SAINT*; program(s) used to solve structure: *SHELXS97* (Sheldrick, 2008 ▶); program(s) used to refine structure: *SHELXL97* (Sheldrick, 2008 ▶); molecular graphics: *SHELXTL* (Sheldrick, 2008 ▶); software used to prepare material for publication: *SHELXTL*.

## Supplementary Material

Crystal structure: contains datablocks global, I. DOI: 10.1107/S1600536810027236/lh5081sup1.cif

Structure factors: contains datablocks I. DOI: 10.1107/S1600536810027236/lh5081Isup2.hkl

Additional supplementary materials:  crystallographic information; 3D view; checkCIF report

## Figures and Tables

**Table:** 

Ni1—O1	1.830 (3)
Ni1—N1	1.846 (4)
Ni1—N3	1.876 (4)
Ni1—N2	1.949 (4)

**Table:** 

O1—Ni1—N1	94.39 (16)
O1—Ni1—N3	89.12 (16)
N1—Ni1—N3	176.32 (19)
O1—Ni1—N2	175.67 (17)
N1—Ni1—N2	87.39 (19)
N3—Ni1—N2	89.03 (19)

**Fig. 1.**
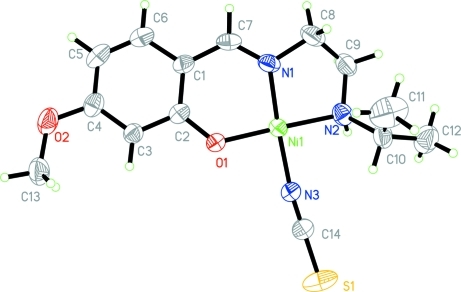
The molecular structure of the title complex, showing 30% probability displacement ellipsoids and the atom-numbering scheme.

## supplementary crystallographic information

### Comment

Schiff bases have often been used as chelating ligands in coordination chemistry (Hamaker *et al.*, 2010; Wang *et al.*, 2010; Mirkhani *et al.*, 2010; Liu & Yang, 2009). A great number of complexes with Schiff bases have been reported for their interesting structures and applications (Keypour *et al.*, 2009; Adhikary *et al.*, 2009; Peng *et al.*, 2009). We report here the crystal structure of the title complex.

The Ni^II^ ion in the title complex is four-coordinated by one phenolate O atom, one imine N atom, and one amine N atom of a Schiff base ligand, and by one N atom of a thiocyanate ligand, forming a slightly distorted square planar geometry (Fig. 1). The bond lengths (Table 1) involving the Ni atom are comparable to those observed in similar nickel complexes (Wang & Wei, 2006; Wang, 2007).

### Experimental

4-Methoxysalicylaldehyde (0.1 mmol, 15.2 mg) and *N*-isopropylethane-1,2-diamine (0.1 mmol, 10.2 mg) were mixed and stirred in methanol (10 ml) for 30 min. Then a methanol solution (5 ml) of nickel nitrate (0.1 mmol, 29.1 mg) was added to the mixture. The final mixture was stirred for another 30 min to give a red solution. Single crystals suitable for X-ray diffraction were obtained by slow evaporation of the solution at room temperature.

### Refinement

Atom H2 was located from a difference Fourier map and refined with an N—H distance restraint of 0.90 (1) Å and U_iso_(H) = 0.08Å^2^. Other H atoms were positioned geometrically (C—H = 0.93–0.97 Å) and refined using a riding model, with with *U*_iso_(H) = 1.2*U*_eq_(C) and 1.5*U*_eq_(C_methyl_). Rotating models were used for the methyl groups.

### Crystal data

**Table:**

[Ni(C_13_H_19_N_2_O_2_)(NCS)]	*F*(000) = 736
*M**_r_* = 352.09	*D*_x_ = 1.461 Mg m^−^^3^
Monoclinic, *P*2_1_/*c*	Mo *K*α radiation, λ = 0.71073 Å
Hall symbol: -P 2ybc	Cell parameters from 2662 reflections
*a* = 12.5653 (10) Å	θ = 2.5–25.3°
*b* = 11.5197 (9) Å	µ = 1.35 mm^−^^1^
*c* = 12.6916 (10) Å	*T* = 298 K
β = 119.393 (4)°	Block, red
*V* = 1600.6 (2) Å^3^	0.25 × 0.23 × 0.22 mm
*Z* = 4	

### Data collection

**Table:**

Bruker APEXII CCD area-detector diffractometer	3458 independent reflections
Radiation source: fine-focus sealed tube	2494 reflections with *I* > 2σ(*I*)
graphite	*R*_int_ = 0.026
ω scans	θ_max_ = 27.0°, θ_min_ = 1.9°
Absorption correction: multi-scan (*SADABS*; Sheldrick, 1996)	*h* = −15→16
*T*_min_ = 0.729, *T*_max_ = 0.756	*k* = −14→12
9225 measured reflections	*l* = −16→15

### Refinement

**Table:**

Refinement on *F*^2^	Primary atom site location: structure-invariant direct methods
Least-squares matrix: full	Secondary atom site location: difference Fourier map
*R*[*F*^2^ > 2σ(*F*^2^)] = 0.058	Hydrogen site location: inferred from neighbouring sites
*wR*(*F*^2^) = 0.160	H atoms treated by a mixture of independent and constrained refinement
*S* = 1.07	*w* = 1/[σ^2^(*F*_o_^2^) + (0.0583*P*)^2^ + 2.8719*P*] where *P* = (*F*_o_^2^ + 2*F*_c_^2^)/3
3458 reflections	(Δ/σ)_max_ < 0.001
196 parameters	Δρ_max_ = 0.75 e Å^−^^3^
1 restraint	Δρ_min_ = −0.60 e Å^−^^3^

### Special details

**Table:**

Geometry. All esds (except the esd in the dihedral angle between two l.s. planes) are estimated using the full covariance matrix. The cell esds are taken into account individually in the estimation of esds in distances, angles and torsion angles; correlations between esds in cell parameters are only used when they are defined by crystal symmetry. An approximate (isotropic) treatment of cell esds is used for estimating esds involving l.s. planes.
Refinement. Refinement of F^2^ against ALL reflections. The weighted R-factor wR and goodness of fit S are based on F^2^, conventional R-factors R are based on F, with F set to zero for negative F^2^. The threshold expression of F^2^ > 2sigma(F^2^) is used only for calculating R-factors(gt) etc. and is not relevant to the choice of reflections for refinement. R-factors based on F^2^ are statistically about twice as large as those based on F, and R- factors based on ALL data will be even larger.

### Fractional atomic coordinates and isotropic or equivalent isotropic displacement parameters (Å^2^)

**Table:**

	*x*	*y*	*z*	*U*_iso_*/*U*_eq_	
Ni1	0.94253 (5)	0.61180 (5)	0.55042 (5)	0.0531 (2)	
N1	0.8398 (4)	0.6742 (3)	0.4000 (3)	0.0593 (10)	
N2	1.0753 (4)	0.6959 (4)	0.5468 (4)	0.0646 (11)	
N3	1.0554 (4)	0.5515 (4)	0.7021 (4)	0.0636 (11)	
O1	0.8235 (3)	0.5227 (3)	0.5535 (3)	0.0610 (8)	
O2	0.4509 (3)	0.3133 (4)	0.4042 (3)	0.0836 (12)	
S1	1.24390 (18)	0.4763 (2)	0.92183 (14)	0.1086 (7)	
C1	0.6645 (4)	0.5528 (4)	0.3496 (4)	0.0545 (11)	
C2	0.7132 (4)	0.5012 (4)	0.4638 (4)	0.0530 (11)	
C3	0.6407 (4)	0.4205 (4)	0.4841 (4)	0.0560 (11)	
H3	0.6705	0.3869	0.5600	0.067*	
C4	0.5259 (5)	0.3910 (5)	0.3922 (5)	0.0639 (13)	
C5	0.4792 (5)	0.4400 (6)	0.2774 (5)	0.0773 (16)	
H5	0.4026	0.4186	0.2152	0.093*	
C6	0.5467 (5)	0.5184 (5)	0.2584 (5)	0.0713 (15)	
H6	0.5149	0.5517	0.1821	0.086*	
C7	0.7299 (5)	0.6411 (4)	0.3259 (4)	0.0621 (13)	
H7	0.6899	0.6777	0.2510	0.075*	
C8	0.8943 (6)	0.7672 (5)	0.3645 (5)	0.0822 (17)	
H8A	0.8816	0.8416	0.3925	0.099*	
H8B	0.8581	0.7700	0.2772	0.099*	
C9	1.0311 (6)	0.7397 (6)	0.4236 (5)	0.0834 (18)	
H9A	1.0448	0.6818	0.3760	0.100*	
H9B	1.0758	0.8093	0.4262	0.100*	
C10	1.1434 (5)	0.7834 (5)	0.6505 (5)	0.0723 (15)	
H10	1.1569	0.7437	0.7243	0.087*	
C11	1.0702 (9)	0.8835 (7)	0.6392 (8)	0.131 (3)	
H11A	1.1127	0.9315	0.7096	0.196*	
H11B	0.9938	0.8590	0.6317	0.196*	
H11C	1.0554	0.9269	0.5686	0.196*	
C12	1.2673 (6)	0.8053 (8)	0.6660 (6)	0.107 (2)	
H12A	1.2595	0.8362	0.5923	0.161*	
H12B	1.3122	0.7338	0.6854	0.161*	
H12C	1.3099	0.8601	0.7303	0.161*	
C13	0.4892 (6)	0.2617 (6)	0.5198 (6)	0.093 (2)	
H13A	0.5602	0.2146	0.5421	0.139*	
H13B	0.4246	0.2142	0.5159	0.139*	
H13C	0.5086	0.3216	0.5790	0.139*	
C14	1.1335 (5)	0.5202 (5)	0.7934 (5)	0.0619 (12)	
H2	1.138 (3)	0.645 (4)	0.570 (5)	0.080*	

### Atomic displacement parameters (Å^2^)

**Table:**

	*U*^11^	*U*^22^	*U*^33^	*U*^12^	*U*^13^	*U*^23^
Ni1	0.0558 (4)	0.0484 (3)	0.0500 (3)	−0.0051 (3)	0.0219 (3)	0.0044 (3)
N1	0.074 (3)	0.046 (2)	0.053 (2)	0.0041 (19)	0.028 (2)	0.0084 (17)
N2	0.077 (3)	0.053 (2)	0.072 (3)	−0.005 (2)	0.043 (2)	−0.005 (2)
N3	0.057 (2)	0.064 (3)	0.056 (2)	−0.006 (2)	0.018 (2)	0.006 (2)
O1	0.0469 (17)	0.074 (2)	0.0515 (17)	−0.0055 (16)	0.0156 (14)	0.0116 (16)
O2	0.065 (2)	0.101 (3)	0.075 (2)	−0.028 (2)	0.0270 (19)	−0.026 (2)
S1	0.1005 (13)	0.1439 (17)	0.0576 (8)	0.0531 (12)	0.0204 (8)	0.0087 (10)
C1	0.049 (2)	0.061 (3)	0.050 (2)	0.011 (2)	0.021 (2)	0.004 (2)
C2	0.048 (2)	0.058 (3)	0.052 (2)	0.008 (2)	0.023 (2)	−0.006 (2)
C3	0.052 (3)	0.063 (3)	0.051 (2)	−0.003 (2)	0.024 (2)	−0.002 (2)
C4	0.053 (3)	0.073 (3)	0.064 (3)	−0.005 (3)	0.028 (2)	−0.020 (3)
C5	0.052 (3)	0.097 (4)	0.061 (3)	0.005 (3)	0.011 (2)	−0.016 (3)
C6	0.063 (3)	0.084 (4)	0.053 (3)	0.009 (3)	0.017 (2)	0.005 (3)
C7	0.071 (3)	0.057 (3)	0.050 (3)	0.022 (2)	0.024 (2)	0.012 (2)
C8	0.112 (5)	0.059 (3)	0.074 (4)	−0.006 (3)	0.044 (3)	0.012 (3)
C9	0.104 (5)	0.076 (4)	0.072 (4)	−0.026 (3)	0.044 (3)	0.004 (3)
C10	0.082 (4)	0.058 (3)	0.078 (3)	−0.021 (3)	0.040 (3)	−0.010 (3)
C11	0.164 (8)	0.104 (6)	0.106 (6)	0.043 (6)	0.052 (5)	−0.027 (5)
C12	0.086 (5)	0.146 (7)	0.093 (5)	−0.027 (5)	0.047 (4)	−0.015 (5)
C13	0.086 (4)	0.110 (5)	0.085 (4)	−0.042 (4)	0.044 (4)	−0.022 (4)
C14	0.064 (3)	0.063 (3)	0.059 (3)	0.002 (2)	0.030 (3)	−0.006 (2)

### Geometric parameters (Å, °)

**Table:**

Ni1—O1	1.830 (3)	C5—C6	1.340 (8)
Ni1—N1	1.846 (4)	C5—H5	0.9300
Ni1—N3	1.876 (4)	C6—H6	0.9300
Ni1—N2	1.949 (4)	C7—H7	0.9300
N1—C7	1.290 (6)	C8—C9	1.534 (8)
N1—C8	1.457 (7)	C8—H8A	0.9700
N2—C9	1.469 (7)	C8—H8B	0.9700
N2—C10	1.541 (6)	C9—H9A	0.9700
N2—H2	0.91 (5)	C9—H9B	0.9700
N3—C14	1.148 (6)	C10—C11	1.436 (9)
O1—C2	1.315 (5)	C10—C12	1.492 (8)
O2—C4	1.361 (6)	C10—H10	0.9800
O2—C13	1.430 (7)	C11—H11A	0.9600
S1—C14	1.618 (5)	C11—H11B	0.9600
C1—C2	1.400 (6)	C11—H11C	0.9600
C1—C6	1.416 (7)	C12—H12A	0.9600
C1—C7	1.429 (7)	C12—H12B	0.9600
C2—C3	1.411 (7)	C12—H12C	0.9600
C3—C4	1.380 (7)	C13—H13A	0.9600
C3—H3	0.9300	C13—H13B	0.9600
C4—C5	1.395 (8)	C13—H13C	0.9600
			
O1—Ni1—N1	94.39 (16)	C1—C7—H7	117.4
O1—Ni1—N3	89.12 (16)	N1—C8—C9	106.3 (4)
N1—Ni1—N3	176.32 (19)	N1—C8—H8A	110.5
O1—Ni1—N2	175.67 (17)	C9—C8—H8A	110.5
N1—Ni1—N2	87.39 (19)	N1—C8—H8B	110.5
N3—Ni1—N2	89.03 (19)	C9—C8—H8B	110.5
C7—N1—C8	119.3 (4)	H8A—C8—H8B	108.7
C7—N1—Ni1	126.5 (4)	N2—C9—C8	109.8 (5)
C8—N1—Ni1	114.2 (4)	N2—C9—H9A	109.7
C9—N2—C10	116.5 (4)	C8—C9—H9A	109.7
C9—N2—Ni1	108.3 (3)	N2—C9—H9B	109.7
C10—N2—Ni1	115.3 (3)	C8—C9—H9B	109.7
C9—N2—H2	113 (4)	H9A—C9—H9B	108.2
C10—N2—H2	97 (4)	C11—C10—C12	116.9 (7)
Ni1—N2—H2	106 (4)	C11—C10—N2	112.3 (5)
C14—N3—Ni1	173.1 (4)	C12—C10—N2	109.2 (5)
C2—O1—Ni1	127.7 (3)	C11—C10—H10	105.8
C4—O2—C13	119.0 (4)	C12—C10—H10	105.8
C2—C1—C6	118.6 (5)	N2—C10—H10	105.8
C2—C1—C7	121.1 (4)	C10—C11—H11A	109.5
C6—C1—C7	120.3 (4)	C10—C11—H11B	109.5
O1—C2—C1	123.8 (4)	H11A—C11—H11B	109.5
O1—C2—C3	117.8 (4)	C10—C11—H11C	109.5
C1—C2—C3	118.4 (4)	H11A—C11—H11C	109.5
C4—C3—C2	120.6 (5)	H11B—C11—H11C	109.5
C4—C3—H3	119.7	C10—C12—H12A	109.5
C2—C3—H3	119.7	C10—C12—H12B	109.5
O2—C4—C3	124.2 (5)	H12A—C12—H12B	109.5
O2—C4—C5	115.1 (5)	C10—C12—H12C	109.5
C3—C4—C5	120.8 (5)	H12A—C12—H12C	109.5
C6—C5—C4	118.8 (5)	H12B—C12—H12C	109.5
C6—C5—H5	120.6	O2—C13—H13A	109.5
C4—C5—H5	120.6	O2—C13—H13B	109.5
C5—C6—C1	122.8 (5)	H13A—C13—H13B	109.5
C5—C6—H6	118.6	O2—C13—H13C	109.5
C1—C6—H6	118.6	H13A—C13—H13C	109.5
N1—C7—C1	125.2 (4)	H13B—C13—H13C	109.5
N1—C7—H7	117.4	N3—C14—S1	179.7 (6)
